# Mgm101: A double-duty Rad52-like protein

**DOI:** 10.1080/15384101.2016.1231288

**Published:** 2016-09-16

**Authors:** Jana Rendeková, Thomas A. Ward, Lucia Šimoničová, Peter H. Thomas, Jozef Nosek, Ľubomír Tomáška, Peter J. McHugh, Miroslav Chovanec

**Affiliations:** a Department of Genetics, Cancer Research Institute, Biomedical Research Center, Slovak Academy of Science, Bratislava, Slovak Republic; b Department of Oncology, Weatherall Institute of Molecular Medicine, University of Oxford, John Radcliffe Hospital, Oxford, UK; c Department of Genetics, Faculty of Natural Sciences, Comenius University, Bratislava, Slovakia; d Department of Biochemistry, Faculty of Natural Sciences, Comenius University, Bratislava, Slovakia

**Keywords:** DNA interstrand cross-link repair, Fanconi anemia, Mgm101, mitochondrial DNA, Rad52, telomere, yeast

## Abstract

Mgm101 has well-characterized activity for the repair and replication of the mitochondrial genome. Recent work has demonstrated a further role for Mgm101 in nuclear DNA metabolism, contributing to an S-phase specific DNA interstrand cross-link repair pathway that acts redundantly with a pathway controlled by Pso2 exonuclease. Due to involvement of FANCM, FANCJ and FANCP homologues (Mph1, Chl1 and Slx4), this pathway has been described as a Fanconi anemia-like pathway. In this pathway, Mgm101 physically interacts with the DNA helicase Mph1 and the MutSα (Msh2/Msh6) heterodimer, but its precise role is yet to be elucidated. Data presented here suggests that Mgm101 functionally overlaps with Rad52, supporting previous suggestions that, based on protein structure and biochemical properties, Mgm101 and Rad52 belong to a family of proteins with similar function. In addition, our data shows that this overlap extends to the function of both proteins at telomeres, where Mgm101 is required for telomere elongation during chromosome replication in *rad52* defective cells. We hypothesize that Mgm101 could, in Rad52-like manner, preferentially bind single-stranded DNAs (such as at stalled replication forks, broken chromosomes and natural chromosome ends), stabilize them and mediate single-strand annealing-like homologous recombination event to prevent them from converting into toxic structures.

## Introduction

*MGM101* (for Mitochondrial Genome Maintenance) was originally identified in a screen for nuclear genes required for maintenance of functional (ρ^+^) mitochondrial DNA (mtDNA) in baker's yeast *Saccharomyces cerevisiae*. The *MGM101* gene, identified by complementation of the temperature sensitive *mgm101-1*^*ts*^ mutant strain,^1^ encodes a 30 kDa DNA-binding protein^2^ that associates with oxidatively damaged mtDNA. Cells lacking functional Mgm101 are sensitive to a variety of DNA damaging agents (ultraviolet light, γ-rays and hydrogen peroxide) under semi-permissive conditions, indicating that Mgm101 participates in the repair of damaged mtDNA.^3,4^ Moreover, Mgm101 has also been shown to be a *bona fide* component of mtDNA-protein complexes called mitochondrial nucleoids (mt-nucleoids),^4^ and is a constituent of mitochondrial membrane-associated replisome, suggesting a role in mtDNA replication.^4,5^ It has been demonstrated that *S. cerevisiae* Mgm101 (*Sc*Mgm101) is required for propagation of mtDNA in cells containing wild-type (ρ^+^) and neutral *petite* genomes, as disruption of *MGM101* blocks initiation of mtDNA replication in these cells.^6^ However, hyper-suppressive (HS) ρ^-^ genomes enriched for GC-rich motifs (dubbed *ori/rep*) are stably maintained in mutants lacking a functional copy of *MGM101*, indicating that these mtDNA derivatives use a distinct mechanism for replication initiation.^4,7^ *Sc*Mgm101 can participate in *ori/rep*-independent mtDNA replication and/or repair by mediating formation of recombination intermediates containing 3′ single-stranded DNA (ssDNA) overhangs.^6^ In this process, Mgm101 was suggested to likely cooperate with Mhr1, Rim1 and the Mre11/Rad50/Xrs2 complex to ensure recombination-dependent DNA double-strand break (DSB) repair after oxidative damage to mtDNA.^3^ However, the exact mechanism of the involvement of Mgm101 in mtDNA replication and/or repair remains unclear.

### Evolutionary conservation of Mgm101 and its characteristics

Mgm101 is a poorly conserved eukaryotic protein with homologs found in many fungal lineages, several amoebas, slime molds and marine animals (sponges, cnidarians, placozoans) but not in insects, nematodes or vertebrates.^6,8^ In plants, 2 paralogous organellar DNA-binding proteins, ODB1 and ODB2, were identified^9,10^ that localize to mitochondria and chloroplasts, respectively, and are also found in the cell nucleus.^10^ The sequences of both proteins are distantly related to Mgm101, although they lack a Pfam signature (PF06420) typical of the Mgm101 homologs. ODB1 was shown to be involved in homologous recombination (HR) and DSB repair in the mitochondria of *Arabidopsis thaliana* suggesting that it may represent a functional homolog of Mgm101.^9^

The majority of our current knowledge about Mgm101 functions is derived from studies of the yeast *S. cerevisiae*. In contrast to most of other fungi, *S. cerevisiae* is a *petite*-positive organism tolerating the loss of functional mitochondrial genome, however there is evidence that the mitochondrial functions of Mgm101 do not dramatically differ between *petite*-positive and -negative yeast species. In line with this opinion, it has been shown that the orthologs of Mgm101 from *Kluyveromyces lactis* (*Kl*Mgm101) and *Candida parapsilosis* (*Cp*Mgm101) functionally complement defects in the metabolism of mtDNA associated with the *mgm101-1*^*ts*^ mutant in *S. cerevisiae*.^11,12^

Comparative sequence analysis of Mgm101 orthologs from yeast revealed a highly conserved C-terminus consisting of 165 amino acids (AA). This region of Mgm101 corresponds to its functional core that is composed of 23% positively charged AA residues and that specifically and tightly binds to (negatively charged) DNA. In contrast, the N-terminus of the protein is not conserved. This region contains a cleavable signal for import into mitochondria. In *Sc*Mgm101, the mitochondrial targeting sequence consists of 22 AA.^6^ In addition, the non-conserved N-terminal region of Mgm101 might be responsible for interspecific differences in DNA-binding and/or interactions with other proteins.^6,13^ Experiments with chimeric Mgm101 proteins consisting of N-terminal domain and a conserved core from 2 different species have demonstrated that the N-terminal domain affects the activity of Mgm101 presumably *via* protein oligomerization and/or the stability of oligomers.^12,13^ Hence, it is supposed that large N-terminal domain of *Sc*Mgm101 shares structural flexibility distributed eventually on ring surface of protein.^3^ It was shown that deletion of 98 AA from the N-terminus of the protein does not have any effect on its ability to complement the *mgm101-1*^*ts*^ allele.^6^ Mbantenkhu *et al*. showed that *Sc*Mgm101 preferentially binds to ssDNA compared to double-stranded DNA (dsDNA) and catalyzes the annealing of ssDNA pre-complexed with the mitochondrial ssDNA-binding protein, Rim1.^14^ In addition, structural organization and protein stability of short 32 AA C-terminal region of *Sc*Mgm101 have been analyzed. The C-terminus consists of highly conserved aromatic and basic AA residues that are able to fold into 2 ß-sheets followed by a short unstructured C-terminus.^14^ K253, W257, R259 and Y268 are indispensable for mtDNA maintenance and K251, R252, K260 affect mtDNA stability at non-permissive condition and under oxidative stress. Mutations in positively charged triad of AA (K251-R252-R253) lead to a failure of ssDNA-binding. The maintenance of mtDNA was affected only under oxidative stress in strains carrying alleles of *MGM101* with mutant codons for K251, K252, K260 or Y266. Finally, W257A and R259A substitutions impaired conformation and ring structure. The authors concluded that short C-terminus of *Sc*Mgm101 is essential for ssDNA-binding, oligomeric state and protein stability *in vivo*.^14^

Biochemical analysis revealed that *Sc*Mgm101 consists of 14 homo-oligomeric rings of approximately 200 Å diameter in solution and that binding to ssDNA promotes formation of large condensed helical nucleoprotein filament structure. Moreover, *Sc*Mgm101 with substitutions C216A C217A exhibits destabilized rings responsible for emergence of stable but defective filaments.^3^ Having a β-β-β-α fold structure makes Mgm101 a member of a large family of Rad52-like proteins, including Redß, Erf from bacteriophage λ and P22, RecT from prophage rac and the Sak from ul36 lactophage.^3,15^ These proteins also form homo-oligomers with 14-fold symmetry *in vitro* but functional relevance of this higher order organization is still unknown.^15^ *Sc*Mgm101 (purified in fusion with MBP domain to increase the yield of soluble protein) was shown to form helical filaments with diameter of approximately 20 nm and to possess ssDNA-binding activity *in vitro*.^15^ Nardozzi *et al*. found that the ring structure disappears when *Sc*Mgm101 binds ssDNA.^3^ The monomers of *Sc*Mgm101 were unstable in solution because they aggregate quickly and are toxic for bacterial cells.^15^

A recent study characterized the Mgm101 protein from the yeast *C. parapsilosis*.^12^ Mitochondria of this species contain linear chromosomes terminating with tandem repeat arrays and 5′ ssDNA overhang.^16-19^ These structures (dubbed mitochondrial telomeres; mt-telomeres) are maintained by a recombination-dependent mechanism involving telomeric circles (t-circles) and telomeric loops (t-loops).^20-23^ Detailed biochemical analysis revealed that *Cp*Mgm101 shares many features with *Sc*Mgm101, but these proteins also exhibit several differences. Both proteins form homo-oligomers in solution, bind to ssDNA with a similar affinity and prefer ssDNA over dsDNA substrates. However, *Cp*Mgm101 binds more strongly to dsDNA which can account for its permanent association with mitochondrial nucleoids and it has been demonstrated that upon binding to 5′ ssDNA overhang of a model mt-telomere it generates ring-shaped structures with the DNA being wrapped around the protein. Such differences indicate a specific role in *C. parapsilosis*. In this light, it is interesting that single-strand annealing (SSA) activity of *Cp*Mgm101 can mediate the invasion of the ssDNA overhang into dsDNA region of mt-telomeres resulting in a formation of t-loop structure and the protein binding to a range of replication and recombination intermediates suggests a role in both mtDNA replication and maintenance of mt-telomeres.^12^ A conciliatory model has been proposed for mitochondrial context, in which Mgm101 may promote strand invasion and error-free recombinational repair by an unconventional utilization of its SSA activity. In this model, Mgm101 would coat ssDNA stretches occurring in mtDNA to generate the Mgm101-ssDNA nucleoprotein filaments, which would directly be annealed to homologous ssDNA donor sequences. Hence, although Mgm101 would predominantly have a role in SSA, strand invasion involving events could represent one of the recombination products during mtDNA replication and repair *in vivo*.^14^

### DNA repair functions of Mgm101 in nucleus

**Figure 1.**
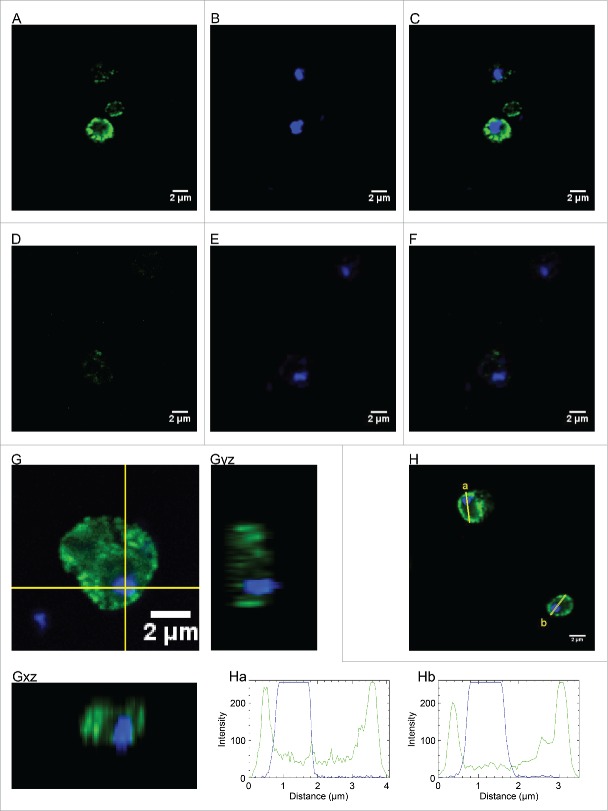
Nuclear localization of Mgm101. *S. cerevisiae* cells with the endogenous *MGM101* locus C-terminally tagged with Myc (strain SSY105, previously described by Ward *et al*.^24^), were fixed with paraformaldehyde and treated with a mouse anti-Myc primary followed by a goat anti-mouse Alexa 488-conjugated antibody. Optimum resolution image z-stacks were collected with a Zeiss LMS510 meta and 100x plan-apochromate objective. A, B and C show the mid-nucleus fluorescent images for Alexa 488nm (green), DAPI (4′,6-diamidine-2′-phenylindole dihydrochloride; blue) and merge, respectively. D, E and F are untagged control cells. G shows a cropped image of a single yeast cell, Gyz and Gxy are the ortholog views through the stack along the lines indicated (yellow). H shows the plots of fluorescent intensity against distance through cells a and b.

In addition to its role in mitochondria, there is increasing evidence that *Sc*Mgm101 also participates in repair of nuclear DNA. Previous studies employing cell fractionation approaches suggest that *Sc*Mgm101 is present in both the nuclear and mitochondrial fractions.^24^ Consistently, an imaging approach supports the presence of diffuse *Sc*Mgm101 within the nucleus (Fig. 1). In cells harboring endogenously Myc-tagged *Sc*Mgm101, confocal z-stack images were sequentially taken to avoid crossover between the DAPI (DNA) and Alexa 488 (*Sc*Mgm101-Myc). Single section images were taken from the middle of the nucleus to reduce the possibility of mitochondrial fluorescence above or below the nucleus appearing to be in the nucleus. Consistent with its localization in the mitochondria and cytoplasm, we observed strong Alexa 488 fluorescence spots and diffuse staining outside the nucleus (Fig. 1A-C). However, diffuse nuclear fluorescence at a similar level to the diffuse cytosolic fluorescence was also observed (Fig. 1A-C, G and H), and in many cases higher concentrations of *Sc*Mgm101-Myc accumulated around the edge of the nucleus (an example can be seen in Fig. 1Ha-Hb). In control cells lacking tagged protein, Alexa 488 staining was barely detectable under the same conditions, excluding the possibility that the observed diffuse fluorescence is due to background levels in the cytoplasm/nucleus (Fig. 1D-F). We also looked at orthogonal views through the nucleus, which show strong cytoplasmic Alexa 488 staining spots above and below but only diffuse *Sc*Mgm101-Myc in the nucleus (Fig. 1Gyz, Gxz). Notably, the induction of DNA damage does not appear to alter the cellular localization of *Sc*Mgm101-Myc (data not shown). *In silico* analysis by PSORT II^25^ and NucPred^26^ identifies putative nuclear localization signals in the core region of Mgm101 homologues. The nuclear functions of *Sc*Mgm101 were deduced from a dual localization of a fraction of the protein and, by inference, its similarity to Rad52:^14,24^ 3 AA residues in Mgm101 (L121, Y139 and K218) are identical with Rad52, and therefore are likely to be required for DNA recombination.^3^ Rad52-mediated HR is highly conserved process that is mainly required for elimination of DSBs and DNA inter-strand crosslinks (ICLs). It is also required for telomerase-independent maintenance of nuclear telomeres.^27-30^ Canonical HR is catalyzed by Rad51 recombinase, the homolog of bacterial RecA, that forms a filament on ssDNA and promotes strand invasion into homologous dsDNA.^31^ Importantly, the SSA activity of Rad52 permits recombination between direct DNA repeats independently of Rad51. Such events, however, are error-prone and generate deletions.^32,33^ It has been suggested that Mgm101 would operate in a SSA-like mechanism similar to Rad59, because Mgm101, as well as other Rad59-like SSA factors, has no large C-terminal domain, which is present in Rad52 and is important for interaction with Rad51.^34^

**Figure 2.**
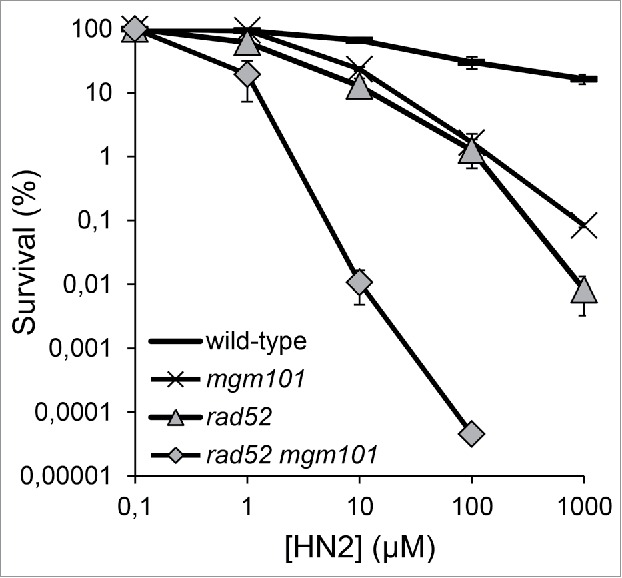
An overlapping role for Mgm101 and Rad52 in the repair of ICLs during S-phase. BY4741 strains disrupted for *MGM101, RAD52* and *MGM101 RAD52* were subject for HN2 treatment following synchronization in S-phase. Analysis was performed as described by Ward *et al*.^24^

*Sc*Mgm101 has been shown to be involved in repair of oxidative DNA damage^4^ that can lead to DSBs. Previous data has shown a crucial role for *Sc*Mgm101 in ICL repair (see below).^24,35^ ICL repair in mammalian cells is governed by Fanconi anaemia (FA) pathway. Defects in this pathway are characterized by bone marrow failure, early onset of leukemia, solid tumors, skeletal abnormal structures (thumb and ulnus), café au lait spots, kidney and urogenital failures and infertility.^36^ We and others have identified a prototypical FA pathway in yeast *S. cerevisiae*,^24,35^ in which putative yeast homologs/orthologs of FANCM (Mph1), FANCJ (Chl1) and FANCP (Slx4), as well as of FANCM-associated histone folding proteins MHF1 (Mhf1) and MHF2 (Mhf2), have been shown to play a fundamental role. FA-like ICL repair further requires Mgm101, the MutSα (Msh2-Msh6) mismatch repair complex, the Smc5-Smc6 complex involved in dynamics and organization of chromosomes, and proliferating cell nuclear antigen (PCNA; Pol30) required for DNA replication.^24,35^ The involvement of Mgm101 in this pathway, as well as its direct interaction with the MutSα complex and Mph1 (recently, the Mph1-Mgm101 physical interaction has also been confirmed by others^37^), highly suggest a role for this protein in metabolism of nuclear DNA after ICL damage. This is also supported by results of a screen of the yeast deletion library that demonstrated *Sc*Mgm101 contributes to the protection of yeast cells against genotoxic effects of formaldehyde, a known protein-DNA crosslinking agent.^38^ We have further examined the role Mgm101 plays in ICL repair, with a particular focus on the putative overlapping function of Mgm101 and Rad52. Disruption of either *MGM101* or *RAD52* lead to an increase in sensitivity to nitrogen mustard (HN2) when cells were synchronized in S-phase (Fig. 2). Disruption of both genes simultaneously caused synergistic increase in sensitivity to this treatment, suggesting that, in this instance, Mgm101 and Rad52 have overlapping activity or are required in parallel pathways.

The FA-like pathway primarily protects stalled replication forks from their collapse into DSBs. Restart of stalled replication forks can be mediated by the Rad5-controlled error-free branch of post-replication repair (PRR) that is independent of Rad6-Rad18-controlled branch of PRR.^39^ In mammals, RAD18 regulates the FA pathway by facilitating the monoubiquitination and chromatin loading of FANCD2-FANCI. Additionally, it contributes to targeting SNM1A to stalled replication forks by monoubiquitinating PCNA and mediating its direct interaction with SNM1A at ICL-stalled replication forks, suggesting that it might be essential for coordinating ICL repair factors acting in parallel or sequentially.^39^ Activation of the RAD18-dependent FA pathway does not appear to be ICL-specific, as it is also involved in response to other types of DNA damage.^39,40^ The Pso2 exonuclease and components of the FA-like pathway act in the processing of an ICL repair intermediate that must be dealt with prior to the initiation of the recombinational stage of ICL repair. This suggests that, similar to human SNM1A,^41^ a tethered cross-linked oligonucleotide is a possible substrate for degradation by Pso2.^24^ We further speculate that in the absence of Pso2 activity the same intermediate is recognized by MutSα, Mph1 and Mgm101. This leads to a recruitment of Exo1 exonuclease, which operates with the same polarity (5′-3′) as Pso2.^42,43^ The resulting degradation of the tethered oligonucleotide allows filling the gap by process of translesion synthesis and recombination. In the absence of Pso2, the replication stalled near the ICL lesion triggers signal for Rad5 to polyubiquitinate PCNA and to recruit the Mph1 helicase. Mgm101, Smc5-Smc6 and Mhf1-Mhf2, likely serve as accessory factors of Mph1 and stabilize the ICL repair intermediate. It has been suggested that during ICL repair Pso2 is dominant over Mph1-Mgm101-MutSα and Exo1 in yeast, as well as Chl1 and Slx4. Despite of absence of the FA core complex in yeast, FA-like pathway represents S-phase specific branch of ICL repair with a functional conservation of the mammalian FA pathway. Mph1/FANCM-mediated regression and stabilization of stalled replication fork is indispensable for efficient step of replication-dependent ICL repair.^39^

### Telomeric functions of Mgm101

There is evidence to suggest that ICL repair factors contribute to the maintenance of nuclear telomeres (for a review see ref. 44). Mammalian nuclear telomeres are composed of long tracts of tandem repeats 5′-TTAGGG-3′ and terminate with 3′ ssDNA overhang. Although it is reminiscent of a typical recombination intermediate, the telomere is protected against inappropriate DNA repair that would result in chromosome fusions. This is achieved by means of a specialized complex of proteins called shelterin that associates with telomeric DNA *via* DNA-protein and protein-protein interactions.^45^ The human shelterin complex prevents chromosome ends from eliciting a DNA damage response, and protects against deleterious degradation or participation in genome-destabilizing recombination or fusion events.^46^ Furthermore, shelterin prevents hyper-resection by components of alternative non-homologous end-joining and HR.^47^

In the nuclei of most eukaryotes, the main mechanism for telomere elongation is mediated by telomerase, a reverse transcriptase carrying its own RNA template that adds telomere repeats *de novo* at chromosome ends.^48,49^ On the other hand, alternative lengthening of telomeres (ALT)^50^ does not rely on telomerase. This mechanism involves HR^29,30,51,52^ and strictly depends on Rad52.^29^ The recombination-dependent telomere maintenance pathways include formation of t-loops and t-circles.^21,53-56^ Intra-chromosomal recombination within telomeric sequences or excision from a t-loop may elicit t-circle formation and, along with shortening of the chromosome ends, it is part of a process known as telomere rapid deletion (TRD).^21,57,58^ There are additional DNA repair factors involved in both telomerase-dependent and -independent maintenance of telomeres such as Mre11 and Exo1 nucleases, as well as Ku heterodimer.^59^

In mammals, FA and a variety of premature aging syndromes are associated with shortened telomeres. Genes that have been shown to be mutated in these diseases participate at least to some extent on DNA damage response and repair, but their precise role in maintaining telomere length is still largely unknown. However, the presence of the SNM1B 5′-3′ exonuclease (Apollo), an ortholog of Pso2/SNM1A, on mammalian telomeres and its interaction with the major telomere-binding protein TRF2^60^ suggest that ICL repair factor(s) could potentially play a role in the maintenance of telomeres. This is supported by an observation that the human FA scaffold protein SLX4 associates not only with DNA repair proteins SLX1, MUS81-EME1 and ERCC1-XPF, but also TRF2, and that this complex primarily associates with long telomeres during late S-phase.^61-63^ The SLX4-containing complex is involved in nucleolytic resolution of telomeric DNA intermediates and disrupting the SLX4-TRF2 interaction results in defective telomere replication.^64^

**Figure 3.**
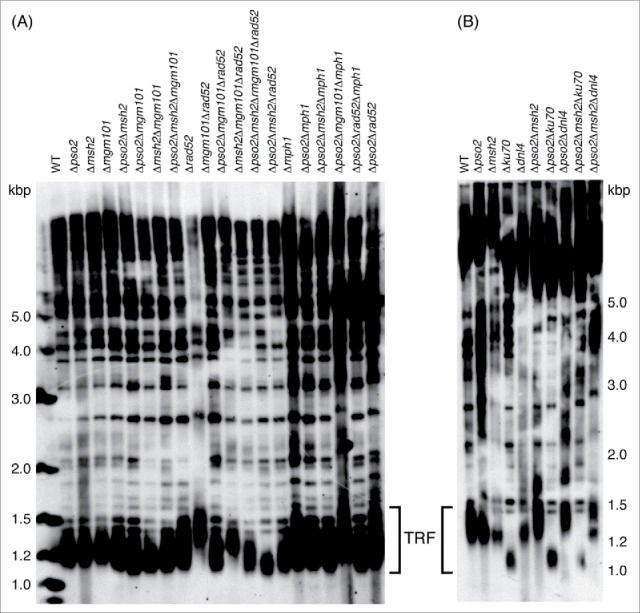
A role of the Pso2-dependent and FA-like pathways in telomere length maintenance in yeast. (A) Pso2-dependent and FA-like pathways in concert with Mgm101 compensate telomeric defects in *rad52* cells. (B) A combination of *pso2* and/or *msh2* with deletion of *YKU70* did not pronounce the shortening of TRFs typical for a *yku70* single mutant. The strains^24^ of *S. cerevisiae* with indicated genotypes were subjected to TRF analysis as described by Šimoničová *et al*.^67^

To determine whether Pso2 and the FA-like pathway in yeast provide a similar contribution to telomere length maintenance, we performed TRF (telomere restriction fragment) analysis in cells deficient for these factors (Fig. 3). Our data suggests that neither Pso2 nor the FA-like pathway alone participates in telomere length maintenance in yeast. However, if both pathways are inactivated, the size distribution of telomeric fragments is modestly changed with a greater enrichment for shorter fragments (Fig. 3A). The possibility of genetic interaction between ICL repair factors and Rad52-dependent recombination event(s) in the process of telomere elongation was also examined. In agreement with previous observations, extensive telomere elongation is observed in *rad52* cells.^65^ Importantly, deletion of either Pso2-dependent or FA-like pathway in *rad52* cells resulted in the recovery of the wild-type telomere length. It is likely that in the absence of the ICL repair factors telomeres are not maintained by ALT mechanism and the shortening of telomeres in the corresponding cells may be caused by decreased levels of telomerase activity or by inefficient recruitment of telomerase to the chromosomal ends. Importantly, a combination of *pso2* and/or *msh2* with the deletion of *YKU70* did not induce a shortening in TRF lengths (Fig. 3B) typical for the *yku70* single mutant,^66^ suggesting that Pso2 and Msh2 act in a different pathway to Ku70.

Double mutant lacking both Mgm101 and Rad52 exhibits normal length of TRFs, suggesting that Mgm101 and Rad52 likely have biochemically very similar (if not identical) functions at telomeres during S-phase. In other words, it appears that Mgm101 is involved in a process of telomere elongation during chromosome replication in *rad52* defective cells. In the light of recent biochemical and structural data,^12^ we speculate that Mgm101 could bind ends of linear DNA molecules, stabilize their structures and mediate SSA-like HR event causing their elongation in cells lacking Rad52. Another possibility is that Mgm101 could be an alternative accessory factor of telomerase which plays a role in telomere length maintenance only in the absence of ALT. Interestingly, *pso2 msh2 mgm101 rad52* quadruple mutant (but neither *pso2 mgm101 rad52* nor *msh2 mgm101 rad52* triple mutant) exhibit shorter telomeres demonstrating that the Pso2-dependent and FA-like pathway cooperate with Mgm101 in telomere maintenance in *rad52* cells. The future experiments aimed at testing a physical association of Mgm101 with telomeric repeats in the wild-type cells as well as *rad52* deletion mutants will address this possibility in a more detail.

## Concluding remarks

Data accumulated over last few years suggests that Mgm101 and Rad52 could have overlapping roles in a yeast cell, at least in the context of nuclear DNA metabolism. This overlap includes a function of both proteins (i) at the site of stalled replication forks where Mgm101 could contribute to repairing DSBs that arise as a consequence of replication fork collapse, and (ii) at telomeres where Mgm101 could be involved in a process of telomere elongation during chromosome replication. We hypothesize that in a Rad52-like manner (biochemically and structurally) Mgm101 could preferably bind ssDNAs, stabilize them and mediate SSA-like HR event to ensure that they are not processed into structures that can be toxic.
